# Efficacy of Vaccination against HPV Infections to Prevent Cervical Cancer in France: Present Assessment and Pathways to Improve Vaccination Policies

**DOI:** 10.1371/journal.pone.0032251

**Published:** 2012-03-12

**Authors:** Laureen Ribassin-Majed, Rachid Lounes, Stephan Clémençon

**Affiliations:** 1 Laboratoire Mathématiques Appliquées à Paris 5, Centre National de la Recherche Scientifique Unité Mixte de Recherche n°8145, Université Paris Descartes, Sorbonne Paris Cité, Paris, France; 2 Laboratoire Traitement et Communication de l'Information, Telecom ParisTech/Centre National de la Recherche Scientifique Unité Mixte de Recherche n°5141, Paris, France; Instituto Butantan, Brazil

## Abstract

**Background:**

Seventy percent of sexually active individuals will be infected with Human Papillomavirus (HPV) during their lifetime. These infections are incriminated for almost all cervical cancers. In France, 3,068 new cases of cervical cancer and 1,067 deaths from cervical cancer occurred in 2005. Two vaccines against HPV infections are currently available and vaccination policies aim to decrease the incidence of HPV infections and of cervical cancers. In France, vaccine coverage has been reported to be low.

**Methods:**

We developed a dynamic model for the heterosexual transmission of Human Papillomavirus types 16 and 18, which are covered by available vaccines. A deterministic model was used with stratification on gender, age and sexual behavior. Immunity obtained from vaccination was taken into account. The model was calibrated using French data of cervical cancer incidence.

**Results:**

In view of current vaccine coverage and screening, we expected a 32% and 83% reduction in the incidence of cervical cancers due to HPV 16/18, after 20 years and 50 years of vaccine introduction respectively. Vaccine coverage and screening rates were assumed to be constant. However, increasing vaccine coverage in women or vaccinating girls before 14 showed a better impact on cervical cancer incidence. On the other hand, performing vaccination in men improves the effect on cervical cancer incidence only moderately, compared to strategies in females only.

**Conclusion:**

While current vaccination policies may significantly decrease cervical cancer incidence, other supplementary strategies in females could be considered in order to improve vaccination efficacy.

## Introduction

Human Papillomavirus infection (HPV) is the most frequent sexually transmitted disease. At least 70 per cent of sexually active men and women are infected with HPV during their life span [1]. Eighty per cent of HPV infections cases are cleared in a few months by the immune system without treatment but in the remaining 20%, infection becomes persistent. One hundred types of HPV have been identified: low risk types, which are responsible for benign anogenital lesions, and high risk types, which can lead to precancerous and cancerous lesions in the cervix. HPV-16 is the most common genotype in developed countries [2], [3].

Epidemiological studies have established a causal relationship between HPV infections and occurrence of cervical cancer [4]. These infections have also been incriminated in anogenital, head and neck cancers, anogenital warts and recurrent respiratory papillomatosis among women and men.

Invasive cervical cancer is the second most common cancer among women worldwide [5]. In France, cervical cancer accounts for about 3,000 new cases per year [6] while a thousand deaths are due to these cancers annually. Moreover, cervical cancers occur frequently in young women [7].

Vaccination against HPV infections represents an effective way to decrease cervical cancer incidence, particularly among young women. Two prophylactic vaccines against HPV infections are available in France and have been found to be highly effective in women who have never been infected with HPV [8].

The permanent Vaccines Advisory Committees (“Comité technique des vaccinations” and “Conseil supérieur d'hygiène publique de France”) recommend vaccinating 14 years old females. Moreover, a catch-up program has been offered to women aged from 15 to 23. Females eligible for the catch-up program either not have been sexually active yet or may report a first sexual relationship that occurred in the year prior to vaccination (Haute Autorité de Santé, HAS). A recent paper has estimated vaccine coverage in France to be low: about 30% of girls aged 14 had been vaccinated with three doses in 2007 and 2008 [9]. Therefore, the potential impact of vaccination has to be assessed considering observed vaccination coverage.

Since the recent introduction of vaccination policies in France, a reduction of cervical cancers and pre-cancerous lesions has been expected. As cervical cancers usually occur 15 years after HPV infections, mathematical models are useful to assess an expected reduction in cancer cases. In these models, vaccine coverage in young women is taken into account.

Various dynamic models have been published to assess the potential impact of HPV vaccination in several countries. Some of them are cohort models [10]–[18] and others are deterministic or hybrid models [19]–[23]. Deterministic models directly allow us to estimate both the direct and indirect (herd immunity) benefits of vaccination [24].

In this paper, we present a deterministic model for the heterosexual transmission of HPV and its progression to cervical lesions and cervical cancer. We estimated the potential impact of vaccination on the reduction of cervical cancer incidence in French women. We first studied the current vaccine coverage to assess vaccine efficacy. Then, vaccine efficacy was assessed in different settings: adding vaccine coverage in boys and young men, achieving higher vaccine coverage in women and adding vaccine coverage in females under 14.

## Methods

### Dynamic Model Structure

**Figure 1:**
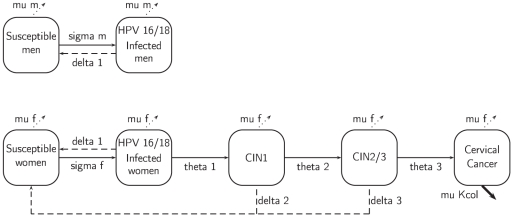
Flow diagram in non-vaccinated population. For one age-group *j* (j = 1…14) and one group of sexual behavior *l* (l = 1…4): Black solid arrows represent infection or progression of the disease; black dotted arrows represent clearance of infection or regression of the disease; gray dotted arrows represent exit of the model (due to death or age >84); bold arrows represent specific mortality due to cervical cancer.

We used Scilab-5.1.1 software (http://www.scilab.org/fr) to design a deterministic model for heterosexual transmission of HPV types 16 and 18. We developed a system of 784 ordinary differential equations (see Table S7). We set the population size in the model to 100,000 individuals, equally divided into females and males. The epidemiologic model simulated heterosexual transmission of HPV-16/18 infections in males and females and progression to CIN1 (cervical intraepithelial neoplasia stage 1), CIN2/3 (cervical intraepithelial neoplasia stage 2/3) and cervical cancer for females (see flow diagram on Figure 1). Description of variables and parameters can be found in Appendix S1 (Table S4).

Fourteen-year-old persons entered the model at a gender-specific and sexual activity-specific rate. Sexually active men and women could be infected with HPV 16/18 if they had had sexual intercourse with infected individuals. Women with CIN1 or CIN2/3 were supposed to be infected with HPV 16/18 and therefore could infect men.

Individuals exited the model by death (age and gender specific using French data) or when they reached the age of 84. An additional death rate for women with cervical cancer was considered [6] (see Table S5).

The heterosexually mixing population was divided into 14 age groups ([14–19], [20–24], [25–29], [30–34], [35–39], [40–44], [45–49], [50–54], [55–59], [60–64], [65–69], [70–74], [75–79], [80–84]) based on age groups of published data on cervical cancer incidence in France [6]. We developed a demographic model [20], [25] which simulated the distribution of French population (see Table S6, Figure S1 and Figure S2). Annual transition rates into age groups were defined by the demographic model (see Appendix S1).

Each age group was divided into 4 levels of sexual behavior. The level of sexual activity was defined by the number of sexual partners in last 12 months (0 sexual partners-including non-sexually-active individuals-, 1 partner, between 2 and 3 partners and more than 4 partners in last year). Results from the French survey on sexual behavior [26] were used to derive the distribution between group of sexual behavior (see Table S1). Mixing between sexual activity groups was quantified by the mixing matrix as described by Garnett and Anderson [27]. The probability for someone from group *l* defining sexual-behavior to form a partnership with someone from group *o* is defined by:
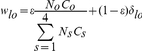
With *N_o_* being the proportion of individuals in sexual-activity group *o*, *c_o_* representing the average number of annual partners in group *o*, the parameter ε described the degree of mixing between sexual activity groups which may vary from fully assortative (ε = 0, when individuals have sexual partners in the same sexual activity class) to fully random (ε = 1). Mixing between sexual activity groups was assumed to be preferentially assortative (ε = 0.4).

### Force of Infection

The force of infection by gender depends on: probabilities of transmission by partnership (not per sex act) from an infected individual to a susceptible (*σ_f_* and *σ_m_*); number of sex partner in last 12 months (*c_l_* = 0, 1, 2–3, +4); proportion of infected individuals in the pool of sexual partner according to their age-group and level of sexual behavior. We developed a mixing matrix *ρ_g,i,k_* appropriate for the sexually active population in France, which gives the proportion of individuals of gender *g*, in age-group *i* who have sexual partners in age-group *k* (see Table S2).

### Transmission model data

In a fitting procedure, we derived the probabilities of transmission of HPV 16/18 (from an infected individual to a susceptible) for both sexes and age-specific progression rates to different stage of cervical disease (CIN1, CIN2/3 and cervical cancer). A set of parameters which matched HPV 16/18 prevalence [28], [29] and age-specific incidences of cervical cancer [30] was selected. As HPV types 16 and 18 are responsible for 70% of all the causes of cervical cancer [31], we multiplied by 0.7 the published French incidence rate of cervical cancer to assess the incidence rate of cervical cancer due to HPV types 16 and 18 (for French women). Regression rates of cervix lesions were defined using literature data [20].

### Vaccine characteristics

We divided the population into vaccinated and unvaccinated categories. Individuals entered the model at 14 years old (being vaccinated or not). Individuals in the youngest age groups ([14]–[19] and [20]–[24]) could be vaccinated after entrance into the model in accordance with the French vaccine program and then moved to vaccinated categories. We considered several vaccination scenarios. Immunity from the vaccine was assumed to be sustained lifelong and vaccine efficacy was assumed to be 90%. In the five scenarios considered, vaccine coverage was assumed to be constant in time. In the first scenario, coverage of vaccination (using 3 doses of vaccines) was set to that observed in France in 2009 [9]: 30% of women aged 14–19 and 10% of women aged 20–24 (Table 1). In the second scenario, we added vaccination coverage in boys and men with similar rates to the women in scenario 1. We thereafter defined hypothetical high coverage in women only (80%) and both genders in scenarios 3 and 4 respectively. Finally, in scenario 5, young girls could be vaccinated before age of 14 with a highest coverage than for girls aged 14 to 24 (50% versus 30%); in this scenario, there was no vaccination in men.

**Table 1:** Scenarios of vaccination considered in simulations.

Vaccine coverage	Scenario 1	Scenario 2	Scenario 3	Scenario 4	Scenario 5
Women					<14: 50%
[14–19]	30%	30%	80%	80%	30%
[20–24]	10%	10%	80%	80%	30%
Men					
[14–19]	-	30%	-	80%	-
[20–24]	-	10%	-	80%	-

### Model validation

To validate the model, we considered the epidemiological data before vaccination introduction and compared it with the steady-state estimates of the deterministic model for non-vaccinated individuals (see Figure S3). Probabilities of transmission of HPV 16/18 (per partnership, from an infected individual to a susceptible one) have been estimated to 0.25 (female) and 0.20 (male).

Among infected females, the rates of progression to various stages of cervical disease (CIN1, CIN2/3 and cervical cancer) were estimated for each age-group (Table S3). We compared the age-specific incidence of cervical cancer predicted by the model with French published data [6], [7]. Incidence rates of cervical cancer in France are estimated using data from cancer registries (www.invs.fr). Seventy percent of cervical cancers are due to HPV types 16/18. Age-specific incidence rates of cervical cancer predicted by the model were similar to the incidence rates of cervical cancers attributed to HPV 16/18 in France within a precision of 10%. Predicted HPV prevalence had a shape and a peak similar to that reported in literature data [28], [29], [32]. Incidence rates of mortality predicted by the model were also close to published rates [7], [33].

### Sensitivity analysis

Sensitivity analyses were conducted to assess the effect of parameter variations on model results. The degree of sexual mixing *ε*_,_ which can vary between 0 (fully assortative) and 1 (fully random), was initially set to 0.4. We tested the effect of a value for *ε* closer to sexual mixing fully random (*ε* = 0.8) on the endemic prevalence of HPV infections in male and female. Although vaccine efficacy was initially set to 90%, in sensitivity analyses, we set vaccine efficacy to 60%. Thus, we studied the impact of vaccination on HPV prevalence and cervical cancer incidence using a low (60%) vaccine efficacy.

## Results

### HPV infection prevalence

Tables 2 and 3 show predicted prevalence of HPV 16/18 infections in each considered scenario of vaccination coverage. All vaccination strategies considered against HPV 16/18 led to a sizeable decrease in prevalence in males and females 20 and 50 years after vaccination introduction (see Figure S4 and Figure S5).

**Table 2:** Prevalence of HPV 16/18 for women in each scenario 10, 20 and 50 years after initiation of vaccination (t = 0).

Prevalence of HPV 16/18 for women	10 years	20 years	50 years
Without vaccination	20.2%	20.2%	20.2%
Scenario 1	14.4% (−28.7%)	9.8% (−51.7%)	2.6% (−87.2%)
Scenario 2	13.7% (−32.2%)	8.31% (−58.9%)	0.85% (−95.8%)
Scenario 3	11.0% (−45.5%)	6.05% (−70.0%)	0.6% (−97.0%)
Scenario 4	10.1% (−50%)	4.69% (−76.8%)	0.09% (−99.6%)
Scenario 5	12.2% (−39.6%)	6.96% (−65.6%)	0.9% (−95.6%)

Vaccine coverage was supposed constant in each scenario. In parentheses, % of reduction in HPV prevalence compared to the case without vaccination.

**Table 3:** Prevalence of HPV 16/18 for men in each scenario 10, 20 and 50 years after initiation of vaccination (t = 0).

Prevalence of HPV 16/18 for men	10 years	20 years	50 years
Without vaccination	19.4%	19.4%	19.4%
Scenario 1	15.1% (−22.2%)	10.9% (−43.8%)	3.7% (−80.9%)
Scenario 2	13.1% (−32.5%)	7.8% (−59.8%)	0.8% (−95.9%)
Scenario 3	12.2% (−37.1%)	7.3% (−62.4%)	1.1% (−94.3%)
Scenario 4	9.6% (−50.5%)	4.3% (−77.8%)	0.1% (−99.5%)
Scenario 5	13.3% (−31.4%)	8.2% (−57.7%)	1.5% (−92.3%)

Vaccine coverage was supposed constant in each scenario. In parentheses, % of reduction in HPV prevalence compared to the case without vaccination.

There was a modest effect on the prevalence of HPV infections when scenarios included vaccination coverage in men and women compared to those considering vaccination coverage only in females. For instance, in France, current vaccination coverage among females lead to a 52% reduction of HPV infection prevalence 20 years after the introduction of the vaccine, while adding vaccination with a similar coverage in boys and young men yielded a 59% reduction in HPV infection prevalence.

Improving vaccine coverage (scenario 3 and 4) decreased HPV prevalence more importantly; in addition, the impact of the vaccines was observed earlier in time.

Scenario 5, which considered an additional vaccination of girls before 14, yielded a better impact on HPV prevalence among females than scenario 2. This scenario considered vaccination with a similar coverage in males and females aged 14 to 24.

The deterministic model that we developed takes into account the reduction of male HPV 16/18 prevalence due to female vaccination. Table 3 shows the expected reduction in male prevalence for each scenario. The reduction of HPV prevalence in men was similar to that in women in case of males and females vaccination (in scenarios 2 and 4).

All in all, scenarios that considered vaccination only in females had a lower impact on the reduction of HPV prevalence in males compared to females.

### Cervical cancer (Table 4)

**Table 4:** Incidence of cervical cancer due to HPV 16/18 in French women in each scenario 10, 20 and 50 years after initiation of vaccination (t = 0).

Time after introduction of vaccine	10 years	20 years	50 years	100 years
Without vaccination	9.6	9.6	9.6	9.6
Scenario 1	8.9 (−7.3%)	6.5 (−32.3%)	1.6 (−83%)	0.5 (−94.8%)
Scenario 2	8.7 (−9.4%)	6.0 (−37.5%)	0.8 (−91.7%)	0.02 (−99.8%)
Scenario 3	8.1 (−15.6%)	4.8 (−50%)	0.5 (−94.8%)	0.05 (−99.5%)
Scenario 4	7.9 (−17.7%)	4.2 (−56.3%)	0.2 (−97.9%)	<10^−5^ (−100%)
Scenario 5	8.5 (−11.5%)	5.3 (−44.8%)	0.7 (−92.7%)	0.07 (−99.3%)

Vaccine coverage was supposed constant in each scenario. In parentheses, % of reduction in cervical cancer incidence compared to the case without vaccination.

Considering current vaccine coverage and screening in France (scenario 1), a 32% reduction of incidence of cervical cancers due to HPV 16/18 may be expected 20 years after the introduction of the vaccine (see Table 4 and Figure S6). A modest reduction may be expected 10 years after vaccine introduction (−7.3%) due to the natural history of cervical cancer: indeed, cervical cancers can occur several decades after HPV infections. Scenario 1 predicted an 83% reduction of cervical cancer cases due to HPV 16/18 at 50 years after vaccine introduction, assuming constant vaccine coverage and screening rates (Table 4).

Adding vaccination among males (scenario 2) to current recommendations (considered in scenario 1) led to a similar reduction in cervical cancer.

Considering a high coverage of vaccination (80% in scenario 3) for women, a halving of numbers of new cervical cancers would be expected 20 years after vaccine introduction. Adding vaccination in men would not lead to a much better reduction in cervical cancer cases (scenario 4).

In scenario 5, females were vaccinated before 14 with a better coverage than females over 14 (50% versus 30%). This scheme led to better results than those from scenario 1 representing current vaccination policy in France. A 45% reduction in cervical cancer incidence could be expected 20 years after vaccine introduction in scenario 5 while scenario 1 yielded a 32% reduction in cervical cancer incidence.

Beyond discrepancies in terms of vaccination impact due to vaccination policies, the scenarios we considered predicted a significant reduction in cervical cancers and infections due to HPV 16/18. A disappearance of cervical cancer due to HPV 16/18 could be expected in a time horizon of 100 years (Table 4). The impact of vaccination on the specific mortality due to cervical cancer appears after decades (Table 5). A reduction by half of annual deaths by cervical cancer could be expected 50 years after the initiation of the vaccine.

**Table 5:** Expected Diminution of number of deaths (per year) due to cervical cancer after introduction of vaccine compared to number of deaths without vaccine.

Time after introduction of vaccine	20 years	50 years	100 years
Scenario 1	−5%	−47%	−88%
Scenario 2	−6%	−56%	−97.5%
Scenario 3	−8.84%	−64%	−97.2%
Scenario 4	−10.58%	−71%	−99.4%
Scenario 5	−7.16%	−59%	−96.17%

### Sensitivity analyses

In sensitivity analysis, we tested the impact of an increase of the sexual mixing parameter that corresponded to an augmentation of random mixing. A high value of the sexual mixing parameter (*ε* = 0.8) led to a 2% reduction of endemic prevalence of HPV infections in males and females compared to the initial value of *ε* (0.4).

A lower efficacy of vaccine (60%) reduced the impact on HPV prevalence and cervical cancer incidence. For instance, using scenario 1, model predictions yielded 50 years after introduction of vaccine a 25% higher HPV prevalence in females and a two-fold increase of cervical cancer incidence compared to scenario 1 with a 90% efficacy of vaccine.

## Discussion

We developed a dynamical model for HPV transmission in a heterosexual population to assess the impact of vaccination against HPV infections on the incidence of cervical cancer in France. We considered current vaccination policies and compared several vaccination scenarios. We confirmed the effectiveness of vaccination against HPV to prevent infections and cervical cancers using deterministic modeling [19], [20], [21], [23]. However, the impact of vaccination differed according to the various scenarios considered in our paper. Vaccination coverage among young females under 14 and higher vaccination coverage in currently targeted females (in France) improved the prevention of infections and cervical cancers due to HPV 16/18 significantly.

The use of a deterministic model in our study allowed us to take into account herd immunity, which corresponds to a decrease of HPV 16/18 infections and cervical cancers in non-vaccinated subpopulations of females due to vaccination coverage of other individuals.

In the fitting procedure, probabilities of HPV 16/18 transmission for women and men were estimated to be of 0.25 and 0.20 respectively. The values that were yielded from our model are close to published estimates from fitted deterministic models for HPV [19], [23]: in the susceptible-infected-susceptible (SIS) deterministic model developed by Taira et al [23], age-specific probabilities ranged from 0.15 to 0.35.

Screening of precancerous lesions of the cervix and cervical cancer using cervical smear tests is recommended in France. Screening coverage has been estimated to be 58.7% in women of 25 to 65 years old in 2005; however, significant discrepancies were observed between age-groups [34]. Screening was not directly integrated in our model as we did not stratify on screening status. Some authors distinguished between regularly screened women and never-screened women [20]. Nonetheless, epidemiological data regarding screening of cervical cancer and cervix precancerous lesions are limited in France. Therefore, our model was fitted on incidence of cervical cancer in France and considered regression of cervix precancerous lesions due to spontaneous regression or treatment after screening. Screening represents a complementary tool to prevent cervical cancers, especially in case of precancerous lesions due to other high-risk HPV types than 16/18 whereas current available vaccines protect against HPV16/18, which are responsible for 70% of cervical cancers.

The study could not take into consideration future changes in screening programs, using new screening policies or new screening technologies on the market. In France, in 2010 Public Health Agency (HAS) proposed to organize cervical cancer screening to improve screening coverage. Moreover, use of new screening tools may also modify current epidemiological data on HPV infections and related cervical cancers. In the USA, the ACOG guidelines (2009) and the American Cancer Society (2003) recommended HPV-plus-pap testing in women aged 30 and older [35]. However, in France, Public Health policies explicitly recommend the use of HPV testing only in cases of abnormal cytology (www.has-sante.fr).

Efficacy of vaccination was assumed to last lifelong. Consequently, we did not include the need for vaccine booster shots in our model. The protective effect of the vaccines is known to last at least several years [36], [37] and the need for boosters is currently unknown.

The sophistication of deterministic models by adding strata corresponding to uncertain data may lead to computational difficulties and increase the uncertainty of outputs and parameters. Thus, screening and use of booster shots were not included in our model.

In sensitivity analyses that investigated the effect of an increase of the sexual mixing parameter (*ε*), we observed a decrease of HPV 16/18 prevalence after using a high value of *ε*. This result is consistent with that reported in Garnett and Anderson paper [27]. We observed that in the presence of frequent completely random sexual mixing (when the value of *ε* is far from the assortative sexual mixing corresponding to *ε* = 0), the infection is less likely to persist endemically [27]. An explanatory mechanism regarding this finding suggests that individuals in the higher sexually active group transmit the infection to individuals in other sexual behavior groups which are less sexually active and less likely to transmit infection.

This analysis shows that the value of *ε* used in our base case modeling is consistent with endemic prevalence of HPV infections. Sexual mixing seems to be preferentially close to assortative case. The modeling of sexual mixing that we developed in this paper could be used in other models corresponding to the French sexual behavior.

Efficacy of both vaccines against HPV has been evaluated in several randomized controlled trials [36]–[38]. While vaccines are highly effective in women who have never been infected with HPV 16/18, efficacy of vaccines could drop in women who have been infected prior to vaccination. We considered in the base case scenario a 90% efficacy of the vaccine against HPV 16/18 infection and 60% in sensitivity analysis. Sensitivity analysis showed that if vaccine efficacy is poor, impact of vaccination on the reduction of diseases linked to HPV 16/18 will be attenuated.

In previous papers assessing the impact of HPV vaccination, assumed vaccine coverage had very high reaching values - between 80% and 100%. Current vaccine coverage in France has been estimated to be low, under 30%, and diminishing over time [9]. Consequently, the impact of vaccination has to be assessed using real life coverage data. In the first scenario, we considered the female vaccine coverage (30%) that is currently reached in France. In the other scenarios of female vaccination (scenarios 3 and 5) we considered realistic alternatives. Finally, we studied scenarios in which males were also vaccinated (scenario 2 and 4).

In the first scenario, we estimated the impact of vaccination on HPV prevalence in male and female, on cervical cancer and on specific mortality due to cervical cancer in the horizon of 10, 20, 50 and 100 years. We considered the vaccine coverage that was observed in France at the beginning of the vaccination campaign (in 2007 and 2008). This scenario predicts a decrease in cervical cancer incidence due to HPV 16/18 by one third 20 years after introduction of vaccine and a halving of deaths by cervical cancer. In this scenario, we considered vaccine coverage to be constant in time. However, female vaccine coverage in France is decreasing. While 33.3% of girls aged 14 in 2007 were vaccinated with 3 doses of the vaccine, only 23.7% and 5.4% of girls aged 14 were vaccinated respectively in 2008 and 2009 [9]. Therefore, the expected effect of the first scenario is probably over-estimated in this paper.

In Scenario 3, we considered a high vaccine coverage for women and young girls (80%), which yielded a better impact of vaccination than scenario 1. In countries where school-based vaccination programs have been implemented (The United Kingdom and Australia), these vaccine coverage rates have been reached [39], [40]. It may be difficult to implement such a program in France, as a previous school-based program of vaccination against hepatitis B failed due to controversies [41].

In scenario 5, young girls could be vaccinated before 14. In Europe, many countries recommend vaccination for females aged 12 [42]. Vaccination of young girls before 14 may improve vaccine coverage and impact of vaccination in France. A better compliance with complete vaccination (using 3 shots) is observed in young females [9] who are little likely to be infected with HPV prior to vaccination. However, the need of booster shots is still unknown [36], [37]. Nevertheless, if the long term efficacy of the vaccine is to be confirmed, vaccination coverage among younger girls will certainly improve the impact of vaccination. The French committee of vaccination recommended the vaccine for girls aged 14 in 2006 [43]. We also investigated the possible vaccination of boys and young men [44]–[47]. In scenarios 2 and 4, we assessed the impact of an additional male vaccination on the reduction of HPV 16/18 prevalence (in males and females) and of cervical cancer incidence in females. Performing vaccination in men improves moderately the effect on HPV 16/18 prevalence and cervical cancer incidence compared to vaccination strategies in females only.

In this paper, we did not assess the impact of vaccination on other cancers or diseases due to HPV 16/18 infections (Recurrent respiratory papillomatosis, cancers of the anus, penis, vagina, vulva, and head and neck). Vaccination against HPV 16/18 infection is likely to reduce incidence of other cancers in the anogenital area in males and female. Reduction of HPV-16/18 prevalence in men due to female vaccination could reduce the incidence of some male cancers. These additional potential benefits of HPV 16/18 vaccination have to be investigated by developing specific dynamic models that consider the natural history of the diseases.

Finally, some recent studies have shown the bivalent vaccine's protective effect against high-risk HPV types, which are not targeted by it (HPV types 31 and 45) [48], [49]. In this case, the model may underestimate the benefit of vaccination on the reduction of cervical cancer incidence as we did not take into account cross-protection in our model.

In conclusion, we developed a deterministic model of HPV heterosexual transmission and progression to cervical cancer to assess the epidemiologic impact of HPV vaccination in France.

While current vaccination policies may decrease cervical cancer incidence in France significantly, other complementary strategies could be employed for females to improve vaccination efficacy.

## Supporting Information

Appendix S1 **Description of the mathematical model.**
(DOC)

Figure S1 **Distribution of women aged 14 to 84.** Data observed in France on 01/01/2006 in blue (www.insee.fr) versus predicted by the demographic model in green.
(EPS)

Figure S2 **Distribution of men aged 14 to 84.** Data observed in France on 01/01/2006 in blue (www.insee.fr) versus predicted by the demographic model in green.
(EPS)

Figure S3 **Number of new cases of cervical cancer due to HPV 16/18 for 100,000 women per year.** Published data in blue [1], predicted by the model in green.
(EPS)

Figure S4 **Female prevalence of HPV 16/18 infection since introduction of vaccination (t = 0) in each scenario.**
(TIF)

Figure S5 **Male prevalence of HPV 16/18 infection since introduction of vaccination (t = 0) in each scenario.**
(TIF)

Figure S6 **Evolution of cervical cancer incidence for French women (number of new diagnosed cases annually per 100,000 women) after introduction of vaccination (t = 0).**
(TIF)

Table S1 **Initial distribution in the model in the 4 sexual-activity groups.**
(DOC)

Table S2 **Mixing matrix between age-group.** Proportion of individuals who have sexual contacts with partners in youngest age-group (<), the same age-group ( = ) or older age group (>).
(DOC)

Table S3 **Model's parameters.**
(DOC)

Table S4 **Description of variables and parameters.**
(DOC)

Table S5 **French mortality rates.**
(DOC)

Table S6 **Distribution of French population aged 14 to 84 (01/01/2006), source: National Institute of Statistics (INSEE).**
(DOC)

Table S7 **Description of matrices used in implementation.**
(DOC)
