# Supplementary material for: Efficacy of Vaccination against HPV Infections to Prevent Cervical Cancer in France: Present Assessment and Pathways to Improve Vaccination Policies
Source: PLoS One. 2012 Mar 12;7(3):e32251. doi: 10.1371/journal.pone.0032251 (PMC3299653; doi:10.1371/journal.pone.0032251)
Supplement: Appendix S1 — Description of the mathematical model. (DOC) [file pone.0032251.s001.doc]

APPENDIX S1

**Demographic model**

Epidemic models of HPV infections take into account the age-structure of population. We developed a demographic model to describe the age-structure of the French population, to assess some demographic parameters and to insert them into the epidemic model. First vaccine which prevents HPV infections has been available in France in 2006. We used the age-structure of French population on 01/01/2006 provided online by the National Institute of Statistics (INSEE website, http://insee.fr) (see Table S5 and Table S6). The demographic model is a modified version of the Hethcote model [4]; adapted by Elbasha et al [5] to have a system of n Ordinary Differential Equations (ODE) which represent the size of n age-group.

We considered 14 age–groups ([14-19], [20-24], [25-29], [30-34], [35-39], [40-44], [45-49], [50-54], [55-59], [60-64], [65-69], [70-74], [75-79], [80-84]).

The system of 28 ODEs describes the distribution of population in each age-group for each gender (see Table S4 for notations):

$$\begin{matrix} dN_{g,1}/dt=\Lambda_{g}-(\mu_{g1}+d_{g1})*N_{g1} \\ dN_{i}/dt=d_{g,(i-1)}*N_{g,(i-1)}-(\mu_{g,i}+d_{g,i})*N_{g,i} \end{matrix}$$

For i=2,…, 14, g=male/female.

Initial numbers of individuals in the first age-group are assessed using equation for each gender (g=male/female):

$$N_{g,1}(0)=N_{g}(1+\sum_{i=1}^{14} \prod_{j=2}^{i} \frac{d_{g,j-1}}{d_{g,j}+\mu_{g,j}})^{-1}$$

Initial numbers of individuals for the other age-groups are computed in an iterative way using equation below (i=2,…, 14; g=male/female):

$$N_{g,i}(0)=\frac{d_{g,i-1}N_{g,i-1}(0)}{d_{g,i}+\mu_{g,i}}$$

**Demographic parameters**

Individuals move between age-groups at transfer rates$d_{g,i}$:

$$d_{g,i}=\frac{\mu_{g,i}}{\exp(band_{i}*\mu_{g,i}-)1}$$

Calibration of demographic model: see Figure S1 and Figure S2.

**Ordinary Differential Equations**

Equations differ in the first age-group and in the others for each compartment. For example, in non-vaccinated compartments of susceptible women:

$\begin{matrix} \frac{dX_{S,j=1,l}}{dt}=(1-\phi_{f,0})\Lambda_{f,l}-\sigma_{f}(\sum_{k=1}^{14} \sum_{o=1}^{4} c_{l}\rho_{f,1,k}\omega_{lo}(\frac{Y_{I,k,o}+W_{I,k,o}}{N_{m,k,o}}))X_{S,j=1,l}+\delta_{1}X_{I,j=1,l}+\delta_{2}X_{CIN1,j=1,l} \\ +\delta_{3}X_{CIN2/3,j=1,l}-(\mu_{f,1}+\phi_{f,1}+d_{f,1})X_{S,j=1,l} \end{matrix}$

For j=2,…,14:

$\begin{matrix} \frac{dX_{S,j,l}}{dt}=d_{f,j-1}X_{S,j-1,l}-\sigma_{f}(\sum_{k=1}^{14} \sum_{o=1}^{4} c_{l}\rho_{f,j,k}(\frac{Y_{I,k,o}+W_{I,k,o}}{N_{m,k,o}}))X_{S,j,l}+\delta_{1}X_{I,j,l}+\delta_{2}X_{CIN1,j,l}+\delta_{3}X_{CIN2/3,j,l} \\ -(\mu_{f,j}+\phi_{f,j}+d_{f,j})X_{S,j,l} \end{matrix}$

The system contains 784 ODEs. We used the matricial notation describes by Capasso [6] to program this non linear system. The system can be written as:

$\frac{dz}{dt}=diag(z)(e+Az)+(F+G+BD)z+c$, t>0

With A, F, G, D, B are matrices of size (784,784), e and c are matrices of size (784, 1) (see Table S7). The element BD *z is the non-linear part of the model.

**Bibliography of the Appendix**

1. Belot A, Grosclaude P, Bossard N, Jougla E, Benhamou E, et al. (2008) Cancer incidence and mortality in France over the period 1980-2005. Rev Epidemiol Sante Publique 56: 159-175.

2. Insinga RP, Dasbach EJ, Elbasha EH (2009) Epidemiologic natural history and clinical management of Human Papillomavirus (HPV) Disease: a critical and systematic review of the literature in the development of an HPV dynamic transmission model. BMC Infect Dis 9: 119.

3. Munoz N, Kjaer SK, Sigurdsson K, Iversen OE, Hernandez-Avila M, et al. (2010) Impact of human papillomavirus (HPV)-6/11/16/18 vaccine on all HPV-associated genital diseases in young women. J Natl Cancer Inst 102: 325-339.

4. Hethcote HW (1997) An age-structured model for pertussis transmission. Math Biosci 145: 89-136.

5. Elbasha EH, Dasbach EJ, Insinga RP (2007) Model for assessing human papillomavirus vaccination strategies. Emerg Infect Dis 13: 28-41.

6. Vincenzo C (2008) Mathematical structures of epidemic systems. New York: Springer.
