# Supplementary figures and images for: Efficacy of Vaccination against HPV Infections to Prevent Cervical Cancer in France: Present Assessment and Pathways to Improve Vaccination Policies
Source: PLoS One. 2012 Mar 12;7(3):e32251. doi: 10.1371/journal.pone.0032251 (PMC3299653; doi:10.1371/journal.pone.0032251)

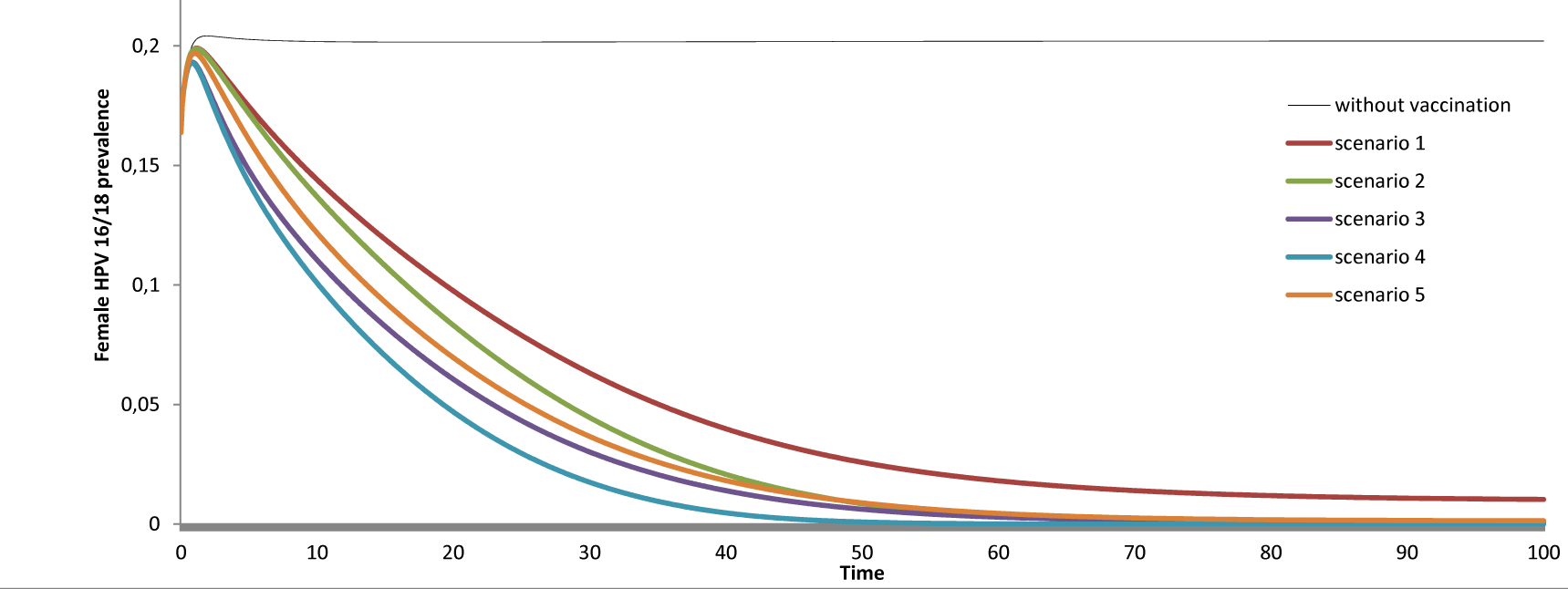

Supplement: Figure S4 — Female prevalence of HPV 16/18 infection since introduction of vaccination (t = 0) in each scenario. (TIF) [file pone.0032251.s005.tif]

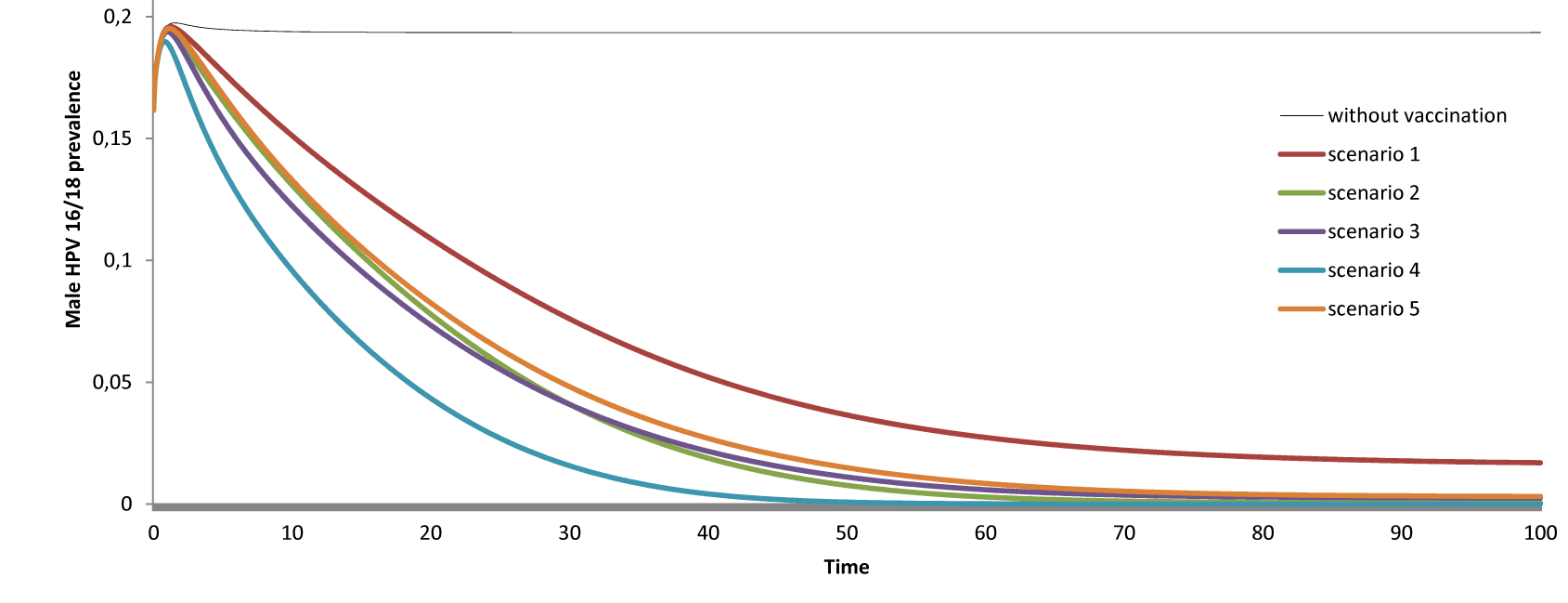

Supplement: Figure S5 — Male prevalence of HPV 16/18 infection since introduction of vaccination (t = 0) in each scenario. (TIF) [file pone.0032251.s006.tif]

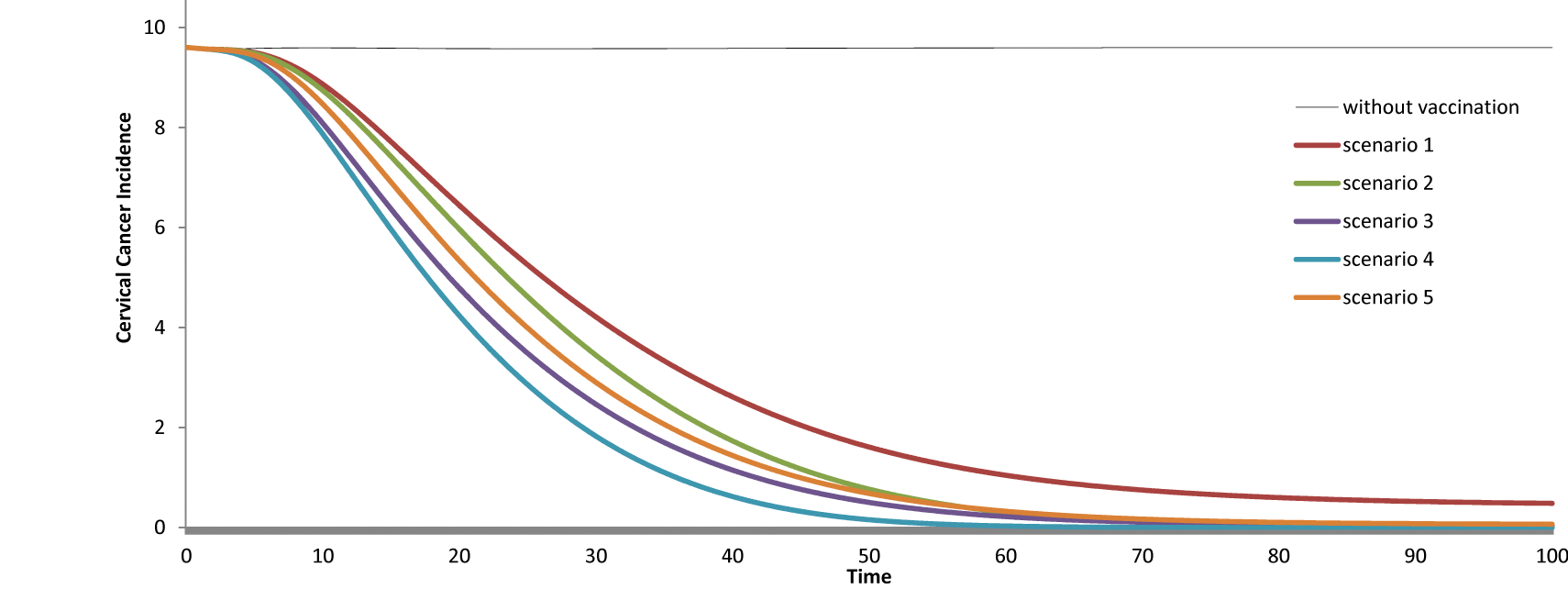

Supplement: Figure S6 — Evolution of cervical cancer incidence for French women (number of new diagnosed cases annually per 100,000 women) after introduction of vaccination (t = 0). (TIF) [file pone.0032251.s007.tif]
