# Supplementary material for: Efficacy of Vaccination against HPV Infections to Prevent Cervical Cancer in France: Present Assessment and Pathways to Improve Vaccination Policies
Source: PLoS One. 2012 Mar 12;7(3):e32251. doi: 10.1371/journal.pone.0032251 (PMC3299653; doi:10.1371/journal.pone.0032251)
Supplement: Table S1 — Initial distribution in the model in the 4 sexual-activity groups. (DOC) [file pone.0032251.s008.doc]

Table S1: Initial distribution in the model in the 4 sexual-activity groups.

|  | Female |  |  |  | Male |  |  |  |
| --- | --- | --- | --- | --- | --- | --- | --- | --- |
| Group of Sexual behavior | 0 | 1 | 2-3 | 4+ | 0 | 1 | 2-3 | 4+ |
| Distribution | 15% | 75%* | 9% | 1% | 15% | 75% | 9% | 1% |

E.g. * 75% of women declared having one sexual partner in last 12 months.
