# Supplementary material for: Efficacy of Vaccination against HPV Infections to Prevent Cervical Cancer in France: Present Assessment and Pathways to Improve Vaccination Policies
Source: PLoS One. 2012 Mar 12;7(3):e32251. doi: 10.1371/journal.pone.0032251 (PMC3299653; doi:10.1371/journal.pone.0032251)
Supplement: Table S2 — Mixing matrix between age-group. Proportion of individuals who have sexual contacts with partners in youngest age-group (<), the same age-group ( = ) or older age group (>). (DOC) [file pone.0032251.s009.doc]

Table S2: Mixing matrix between age-group. Proportion of individuals who have sexual contacts with partners in youngest age-group (<), the same age-group (=) or older age group (>).

|  |  | % Female |  |  | % Male |  |
| --- | --- | --- | --- | --- | --- | --- |
|  | < | $\rho_{f,s,t}$ = | > | < | $\rho_{m,s,t}$ = | > |
| [14-19] | - | 35 | 65 | - | 86 | 14 |
| [20-24] | 5 | 56* | 39 | 25 | 62 | 13 |
| [25-29] | 7 | 46 | 47 | 38 | 46 | 16 |
| [30-34] | 8 | 46 | 46 | 42 | 44 | 14 |
| [35-39] | 15 | 41 | 44 | 45 | 40 | 15 |
| [40-44] | 16 | 44 | 40 | 46 | 44 | 10 |
| [45-49] | 16 | 39 | 45 | 50 | 35 | 15 |
| [50-54] | 13 | 42 | 45 | 45 | 41 | 14 |
| [55-59] | 19 | 42 | 39 | 46 | 46 | 8 |
| [60-64] | 22 | 40 | 38 | 56 | 50 | 7 |
| [65-69] | 17 | 47 | 36 | 53 | 39 | 8 |
| [70-74] | 17 | 47 | 36 | 53 | 39 | 8 |
| [75-79] | 17 | 47 | 36 | 53 | 39 | 8 |
| [80-84] | 17 | 83 | - | 53 | 47 | - |

e.g. *56% of women in [20-24] age-group have contact with men of the same age-group.
