# Supplementary material for: Efficacy of Vaccination against HPV Infections to Prevent Cervical Cancer in France: Present Assessment and Pathways to Improve Vaccination Policies
Source: PLoS One. 2012 Mar 12;7(3):e32251. doi: 10.1371/journal.pone.0032251 (PMC3299653; doi:10.1371/journal.pone.0032251)
Supplement: Table S3 — Model's parameters. (DOC) [file pone.0032251.s010.doc]

Table S3: Model's parameters

| Parameters | female | male | Reference number(s) |
| --- | --- | --- | --- |
| Demographic |  |  |  |
| $N_{g}$size of population for gender g | 50,000 | 50,000 | * |
| Natural history ** |  |  |  |
| Probability of transmission for women $\sigma_{f}$ | 0.25 |  | Calibration |
| Probability of transmission for men $\sigma_{m}$ | 0.20 |  | Calibration |
| Clearance rate for HPV 16/18 ($\delta_{1}$) | 0.83 | 0.83 | [2] |
| Regression rate for CIN1 ($\delta_{CIN1}$) | 0.329 | - | [2] |
| Regression rate for CIN2/3 ($\delta_{CIN2/3}$) | 0.21 | - | [2] |
| Age-specific Progression rates from infected to CIN1 () |  |  | Calibration |
| [14-19] | 0.005 |  |  |
| <19 | 0.05 |  |  |
| Age-specific Progression rates from CIN1 to CIN2/3 ($\theta_{2,j}$) |  |  | Calibration |
| [14-19] | 0.005 |  |  |
| [20-24] | 0.025 |  |  |
| <24 | 0.055 |  |  |
| Age-specific Progression rates from CIN2/3 to cervical cancer ($\theta_{3,j}$) |  |  | Calibration |
| [14-19] | 0.0025 |  |  |
| [20-24] | 0.0125 |  |  |
| [25-29] | 0.025 |  |  |
| [30-34] | 0.022 |  |  |
| [35-39] | 0.022 |  |  |
| [40-44] | 0.025 |  |  |
| [45-49] | 0.02 |  |  |
| [50-54] | 0.02 |  |  |
| [55-59] | 0.02 |  |  |
| [60-64] | 0.02 |  |  |
| [65-69] | 0.02 |  |  |
| [70-74] | 0.02 |  |  |
| [75-79] | 0.022 |  |  |
| [80-84] | 0.022 |  |  |
| Vaccine |  |  |  |
| Vaccine degree of protection $\tau$ | 90% |  | [3] |

*Compartment size enough large to apply a deterministic model

**The natural history parameters are annual transition rates
