# Supplementary material for: Efficacy of Vaccination against HPV Infections to Prevent Cervical Cancer in France: Present Assessment and Pathways to Improve Vaccination Policies
Source: PLoS One. 2012 Mar 12;7(3):e32251. doi: 10.1371/journal.pone.0032251 (PMC3299653; doi:10.1371/journal.pone.0032251)
Supplement: Table S4 — Description of variables and parameters. (DOC) [file pone.0032251.s011.doc]

| Symbol |  | Description |
| --- | --- | --- |
| *g* |  | Gender=male/female |
| *j* |  | Age-group, *j*=1,…,14 |
| *l* |  | Group of sexual behavior, *l*=1,…,4 |
| Variables |  |  |
| Non vaccinated | Vaccinated |  |
| $X_{S,j,l}(t)$ | $V_{S,j,l}(t)$ | Susceptible women, age-group *j*, group of sexual behavior *l* |
| $X_{I,j,l}(t)$ | $V_{I,j,l}(t)$ | Infected women with HPV 16/18 |
| $X_{CIN1,j,l}(t)$ | $V_{CIN1,j,l}(t)$ | Women with CIN1 |
| $X_{CIN2/3,j,l}(t)$ | $V_{CIN2/3,j,l}(t)$ | Women with CIN2/3 |
| $X_{K,j,l}(t)$ | $V_{K,j,l}(t)$ | Women with cervical cancer |
| $Y_{S,j,l}(t)$ | $W_{S,j,l}(t)$ | Susceptible men |
| $Y_{I,j,l}(t)$ | $W_{I,j,l}(t)$ | Infected men with HPV 16/18 |
| Demographic parameters |  |  |
| $\Lambda_{g,l}$ |  | New recruits into the sexually active population (g= male/female) |
| $\mu_{g,j}$ |  | Death or remove rate from the sexually active population in age-group j (g= male/female) |
| $\mu_{K,j}$ |  | Specific mortality rate of cervical cancer in age-group j |
| $N_{g}(t)$ |  | Number of men (g=m) and women (g=f) |
| $N_{g,j}(0)$ |  | Initial number of individuals of gender g in age-group j |
| $band_{j}$ |  | Number of years in age-group j |
| Vaccines Parameters |  |  |
| $\phi_{g,0}$ |  | vaccination rate before 14 |
| $\phi_{g,j}$ |  | vaccination rate in age-group j |
| $\tau$ |  | degree of vaccine protection |
| Sexual behavior |  |  |
| $c_{l}$ |  | Number of sexual partners in last 12 months in group of sexual behavior l |
| $\rho_{g,i,k}$ |  | Proportion of individual of gender g in age-group I who form partnership with individuals of age-group k |

Table S4: Description of variables and parameters
