# Supplementary material for: Efficacy of Vaccination against HPV Infections to Prevent Cervical Cancer in France: Present Assessment and Pathways to Improve Vaccination Policies
Source: PLoS One. 2012 Mar 12;7(3):e32251. doi: 10.1371/journal.pone.0032251 (PMC3299653; doi:10.1371/journal.pone.0032251)
Supplement: Table S5 — French mortality rates. (DOC) [file pone.0032251.s012.doc]

Table S5: French mortality rates

| Age-specific Mortality rates (/1000) | Female | Male | Source |
| --- | --- | --- | --- |
| [14-19] | 0.2 | 0.5 | * |
| [20-24] | 0.3 | 0.9 |  |
| [25-29] | 0.3 | 0.9 |  |
| [30-34] | 0.4 | 1.1 |  |
| [35-39] | 0.7 | 1.5 |  |
| [40-44] | 1.2 | 2.3 |  |
| [45-49] | 1.9 | 4 |  |
| [50-54] | 2.7 | 6.2 |  |
| [55-59] | 3.6 | 8.7 |  |
| [60-64] | 5.1 | 11.9 |  |
| [65-69] | 7.3 | 17.3 |  |
| [70-74] | 16.5 | 34.5 |  |
| [75-79] | 16.5 | 34.5 |  |
| [80-84] | 54 | 87 |  |
| Additional Mortality rates for cervical cancer (/100,000) |  |  | [1] |
| [14-19] | 0 |  |  |
| [20-24] | 0 |  |  |
| [25-29] | 0.3 |  |  |
| [30-34] | 1.1 |  |  |
| [35-39] | 2.5 |  |  |
| [40-44] | 3.7 |  |  |
| [45-49] | 4.9 |  |  |
| [50-54] | 5.1 |  |  |
| [55-59] | 4.5 |  |  |
| [60-64] | 4.7 |  |  |
| [65-69] | 5.3 |  |  |
| [70-74] | 6.7 |  |  |
| [75-79] | 8.9 |  |  |
| [80-84] | 11.6 |  |  |

*source: National Institute of Statistics ([www.**insee**.fr](http://www.insee.fr/))
