# Supplementary material for: Efficacy of Vaccination against HPV Infections to Prevent Cervical Cancer in France: Present Assessment and Pathways to Improve Vaccination Policies
Source: PLoS One. 2012 Mar 12;7(3):e32251. doi: 10.1371/journal.pone.0032251 (PMC3299653; doi:10.1371/journal.pone.0032251)
Supplement: Table S6 — Distribution of French population aged 14 to 84 (01/01/2006), source: National Institute of Statistics (INSEE). (DOC) [file pone.0032251.s013.doc]

Table S6: Distribution of French population aged 14 to 84 (01/01/2006), source: National Institute of Statistics (INSEE).

| Age-group | Number of individuals (%) |  |
| --- | --- | --- |
|  | Female | Male |
| [14-19] | 2,437,642 (9.37%) | 2,539,245 (10.30%) |
| [20-24] | 1,606,562 (6.18%) | 2,028,486 (8.23%) |
| [25-29] | 1,960,605 (7.54%) | 1,929,741 (7.83%) |
| [30-34] | 2,173,881 (8.36%) | 2,140,918 (8.69%) |
| [35-39] | 2,253,084 (8.66%) | 2,211,916 (8.98%) |
| [40-44] | 2,304,938 (8.86%) | 2,236,711 (9.08%) |
| [45-49] | 2,232,055 (8.58%) | 2,142,154 (8.69%) |
| [50-54] | 2,176,491 (8.37%) | 2,079,431 (8.44%) |
| [55-59] | 2,162,610 (8.31%) | 2,086,773 (8.47%) |
| [60-64] | 1,454,492 (5.59%) | 1,373,393 (5.58%) |
| [65-69] | 1,396,503 (5.37%) | 1,221,179 (4.96%) |
| [70-74] | 1,434,064 (5.51%) | 1,123,295 (4.55%) |
| [75-79] | 1,326,806 (5.10%) | 907,401 (3.68%) |
| [80-84] | 1,092,305 (4.20%) | 620,938 (2.51%) |
| All | 24,641,581 (100%) | 26,012,038 (100%) |
