# Supplementary material for: Efficacy of Vaccination against HPV Infections to Prevent Cervical Cancer in France: Present Assessment and Pathways to Improve Vaccination Policies
Source: PLoS One. 2012 Mar 12;7(3):e32251. doi: 10.1371/journal.pone.0032251 (PMC3299653; doi:10.1371/journal.pone.0032251)
Supplement: Table S7 — Description of matrices used in implementation. (DOC) [file pone.0032251.s014.doc]

Table S7: Description of matrices used in implementation**.**

| Matrix | Size | Description |
| --- | --- | --- |
| c | (784,1) | New entrance in the model |
| e | (784,1) | Exit of each compartment by: transfer of age-group, death, progression or regression of infection or disease |
| A | (784,784) | Incident cases of HPV infections which leaves susceptible compartments |
| B | (784,784) | Non constant matrix composed of elements of z |
| D | (784,784) | Incident cases of HPV infections which enter infected compartments |
| F | (784,784) | Entrance in compartments due to progression of disease or clearance of infections |
| G | (784,784) | Entrance in compartments due to transfer between age-groups |
